# Metropolitan School of Dental Science

**Published:** 1860-01

**Authors:** 


### ARTICLE XV.

### *Metropolitan School of Dental Science.*

THIS School was inaugurated on Wednesday, October 5th, in the presence of a large number of the medical and dental profession.

Dr. Richardson, the professor of anatomy and physiology, at the request of his colleagues, delivered the inaugural address, which was listened to with marked attention.

We subjoin a verbatim copy.

### INAUGURAL ADDRESS.

GENTLEMEN.—We have met to-day, to open in formal manner a school for the advancement of the science and art of Dentistry. The undertaking is novel in a double sense. The school is new, and the school is the first of its kind in this kingdom. The history-books tell us of our forefathers, "that cut off by sea from the rest of the world, they became mariners at first from necessity." It may be, in the future, said of the modern dentists, that cut off by custom from the rest of the scientific world, they made scholars at first from necessity; and I trust it will be added in continuance of the argument, that what originally seemed an inconvenience, turned out in the end so much to their advantage that they are acknowledged by all persons to be the undisputed lords of the science ocean.

Were I, in the remarks I have to make, to follow entirely the bent of my own inclinations, I should try to beguile the hour by addressing myself as a teacher to those exclusively whom we hope to see taking permanent possession of these benches; not as listeners to a discourse so transitory as this, and spoken at a time when a warm enthusiam prevails; but as calm, hard-working disciples, determined on one point, that nothing which the dominie knows, and is willing to tell, shall be unlearned. I should like, I say, to make this subject-matter apply especially to these; to point out to them, in so far as my ability may permit, the mental elements required in the course of philosophical labor, the methods of inquiry, the worth of the study, aud the rewards and the happiness resultant.

But inasmuch as I have come here with yet another intention before me, as I stand, for the moment, the representative of those who have projected this school, it is incumbent on me in the first instance to describe, in simple and practical terms, the principles upon which the school is founded, and the character of the teaching which is here to be carried out.

The first intention, then, is to elevate the dental profession, by affording the facilities for a liberal professional education, by asserting, without assumption, the independency of the profession, and by aiming at the establishment of an entire unity, either now or hereafter, amongst its divided brotherhood.

Until within these last few years, dentistry as a particular calling has had no recognition, except if the expression is allowable, in that individualism which has marked it out as a practical science and art, followed by special men since the remote period when Herodotus himself met its practitioners in the Egyptian cities. All else has congregated itself, and claimed recognition; the priest has his order, the barrister his temple, the physician his college, and the merchant his guild. The dentist, isolated by his pursuits as much as the rest, his nothing.

But these last few years have wrought a great change here as abroad. America has now her dental institutions in abundance—the continent its schools—while, in England, where the organization has been last to commence, and commencing has met with unusual, unexpected, and I am right when I say, *unnecessary* difficulties, it has progressed so quickly withal as to have led to the establishment of a college, and of a learned society kindred to others of older growth. These institutions, each alike important in their respective spheres, are by their very existence clear indications of an association of ideas, if not at once of men. But such institutions, though by nature primary and preeminent, in that the manhood and full intellectual capacity of the section they represent is embodied in them, are not complete without a third, viz. one that shall give the ready means for the acquirement of knowledge to the junior or less advanced members of the professional body. An institution that shall induct these into their proper places in the older establishments, to supply as each generation passes to its inevitable rest, another, and another, and another, each one more perfect in knowledge, and reflecting a degree of respect and information equal to the taste, the refinement, and the intelligence of the age. I have now explained the meaning, present and remote, of the Metropolitan School of Dental Science.

To make their exertions the more useful and accessible, the founders of this school have endeavored to take not only a liberal, by what is considered by all men freed from the bias of corporate interest, an advanced position in educational concerns. Guided by the experience, and forewarned by the errors of past educational schemes, they have cast to the winds all fancies about restrictions, preliminaries, curriculums, and prison-like enactments. Believing not only the anatomical truth, that every man has a brain, but the physiological fact also, that every brain is impressionable under sound and useful tuition, they open their doors to every respectable youth or man who, with the self-respect natural to a gentleman, will listen to their teachings. But enforcement there is none. The teacher will lecture under no hard system—the student will learn in liberty. Who is there that wishes not the knowledge for its own sake, and for the certain advantages following its possession? He has no invite here. Or, as the great

English Protector said to his never-beaten volunteers:—

> "I pray consider it. Do I come to tell you that I would tie you to this war? No. But if you can come to prosecute it, prosecute it vigorously, or don't do it at all. I think it my duty to deal plainly; therefore this is what I advise you—that we join together to prosecute it vigorously."

Nor can it be feared, so long as no intermeddling statecraft intervenes, that success is not to follow such liberal enjoyment of educational pursuits. The history of the past, in its triumphs and in its errors, tells us in fact that education is never so ennobled as when it is free, and never so degraded as when it is fettered. The history of medical education is pregnant of this truth. I turn to the time when corporate greed and intolerance laid no hand on medical knowledge, and there are the schools worthy of the name, and there are the teachers of immortal celebrity, and there are the students who rival the masters. I see the school at Padua, and Fabizzio teaching the traveling Englishman the elements of the circulation. I see the school of William Hunter, and William teaching to unrestrainable brother John the rudiments of the classification of the living and the dead creation. In the same free school I see again the said John Hunter instructing the earnest Jenner in that method of research which was afterward to guide him to the great practical discovery of vaccination.

From this I turn to the modern school, with its divided, and divided, and divided schedules,—upon which mere compressed ragstuff all the unity of science is broken—with its three-year formulas, and its shepherd porters counting in the flock to listen unwittingly. And what is the effect of the contrast? Gazing at the one the true lover of science feels a glow, an energy, a soul-lighted enthusiasm, which lifts him, like sweet sounds or the beauty of a sunsetting skyscape, almost out of this little life. Turning to the other the whole dead attraction of earth draws him towards its centre, and so binds him hand and foot, as by magnetic cold, that it were relief to sleep from the picture, though the sleep were jeopardous.

The founders of the present school, as men of the world knowing the limitations of their project, make no pretension to rival the great schools above named. But if not in fame, they would at least, if it were possible, imitate them in that freedom which has so often nursed fame ; and thus striving would leave their endeavors to the consideration of all common-sense men, convinced that they who know most of education as an artistic effort will be the last to criticise.

The plan of instruction instituted in this school next calls for a word of description. It may be that in progress of time the practice of teaching will indicate some modifications or improvements. After the most careful consideration, however, of the requirements of the present day, the faculty of lecturers feel that their design may be considered as accordant with the principles upon which the school is founded, and with its scope as an educational centre for members of a section of the community engaged in a special pursuit.

It has been urged repeatedly that for the necessities of one engaged in the pure practice of dentistry, there are actually required, in addition to a good general education, competent knowledge of but three scientific branches, viz. *anatomy*, *dental surgery* and *pathology* and *dental mechanics*. Grounded well in these three departments, and made directly conversant with them in practice, a dentist, it is argued, possesses all that particular information which the practice of his daily life and the welfare of the public demands.

In this argument there is an undoubted practical force and logic of no mean distinction. That is to say, there is a relationship between pure dentistry and medicine which is best maintained in amity, and with mutual advantage, by such friendly difference in the practical parts of each as shall prevent encroachment on either side, and the jealousy that might follow on such encroachment. In a word, it is felt that the present excellent understanding which obtains between the two professions, and by which they are and always will be equally benefited, is due to the distinctions of practice and of qualification.

In marking out the educational system of the "Metropolitan School of Dental Science," all the strength of this argument has been admitted, and it has been arranged that the three above named departments of science shall stand as the basis of the educational plan.

At the same time a more extended idea of the scientific endowments of the dental profession has been taken. It is assumed that the practitioner in dentistry, engaged in a pursuit that fills up his working hours in special scientific labor, is in a position, without any trespass on other practical pursuits, to devote a learned leisure to the pursuance of those higher branches of science which, unconnected mainly with the bread-earning duties, raise man above the drudgeries of life, and proclaim in demonstrative terms that in his full attributes—derived from a contemplation of the higher branches of natural knowledge—each man partakes of that divinity which once everywhere and even now in barbarous nations is allowed only to enshrine the person of the king. It is assumed that the dental profession has the means, as the will, to pursue in quiet moments these greater problems of the universe ; and it is believed, as indeed it has been shown, that the members of this profession are never themselves so elevated, or their profession through them, as when they take a prominent position in these nobler studies, or sit, as one of them (Professor Bell) now most honorably does, in presidential rule over the followers of a philosophical sect. With these feelings, then, the scope of the school has been enlarged beyond the mere necessaries. There are added to it three distinct studies, which will give the Attic salt to the edibles, will render labor more pleasant, and will open the intelligent mind to thoughts and acts which the longest lifetime enjoys but too briefly. And this leads me to enter in some detail into the subjects which are really to be taught.

We take *anatomy* then, as the first and basic subject, and in so far as time will permit, we shall make every effort to give to this department its due importance. Practical instruction must here of necessity be the secret of success to the teacher, and practical learning for the taught. The student will find in this pursuit the simplest and most satisfactory part of his studies. In this all is straightforward sailing. The acceptance into the volume of the brain of natural records, freed from speculation, and acquired with mathematical precision.

To this anatomy we add *physiology*, or that science that gives vitality to the dead record. Which says of this bone, this muscle, this nerve, in their living time these parts did such and such duties. Without this sister science anatomy were disgusting in its details, and comparatively useless in its results. With it the heaviness of death is past, and the mind, receiving glimpses of those living actions which concern us most intimately, sees open to it a new world of thought, more expansive than is supplied by the mere material universe in its widest representations.

It is wonderful, overpoweringly wonderful, to go with the astronomer in his wanderings. To see him gauge the infinite; to observe him, with the genius of Leverrier, step in mental calculation out of the extremest known range of this system of planetary worlds, and add thereto another world, which has never before been seen, but appears as at his bidding. To see him next hiding himself for a season in the glory of the sun, return and say that between the radiant luminary and the first known planet of the system there exists yet another,

"Lost in the near effulgence of his blaze."

But what of this in its wonder to the still greater wonder which is opened to the philosopher of life? To him who shall read the physiological laws truly; who shall master the dead matter the dissector maps out; to whom shall be revealed the nature of that living force and principle by which the greatest of material problems are wrought out, by which the mind in mastering those problems performs its functions to that end, by which the hand which perpetuates the labor does its task in obedience to the mind?

When, then, we include in our scheme the science of life, we offer to the student the richest branch of natural study open to his intelligence. A work in which he can not only *do* and follow, but *do* and *lead*. A work which, but little understood by the public, claims pupils by the million. A work to the which every simple, well-known thing, every insect, every flower, every man, offers a problem.

To give another and more practical meaning to anatomy and physiology, we shall blend with it, incidentally, a third subject, *pathology*, or the study of disease. It will be obvious that in this direction our attention must be confined chiefly to the pathology of the organs of mastication; but enough will also be supplied to guide the learner to a knowledge of the general and more useful principles of this science.

The second division of our educational scheme is entitled *dental surgery*. In this department the teacher will ask attention to those practical daily duties upon which the science, as a science, mainly rests. There will here be introduced the history of surgical manipulations on the dental organs; the symptoms, course, and results of dental disease. The effects of various agencies, constitutional and local, in the production of such disease. The influence in their turn of these diseases on the body at various periods of life, and an account of those measures, remedial, or preventive, or both, which the most advanced experience shows to be of the greatest service in treatment.

The third subject in our design is *dental mechanics*, or that art, for here dentistry takes in an art of the most simple and beautiful type, which shows how nature may be supplemented, and I say it with all deference to the worthy dame, in some cases surpassed; and again how natural parts may by sundry scrapings and stoppings be restored and preserved. There has been, and if I am not erring still is, an attempt made now and then to ignore this department of dentistry, because it is mechanical. I have never, however, for my part met with any dentist or surgeon-dentist who so far forgot himself, in ignoring it, as to forget the receipts of certain advantages derived from it in the shape of such specimens of fine metals as it delights the masters of Her Majesty's mint to stamp right royally, or of such relics of crisp paper as the little old woman in Threadneedle street chooses to shape into billet-doux for her admiring favorites.

Anyway the founders of the new school have no such scruples, and being themselves (such of them as can practice the art) rather accustomed to find it an essential; and being further assured that he who cannot practice it, or at least superintend it, is but half way towards good or successful practice, they have felt it a duty to make this subject a basic one for the student; and that it may be taught thoroughly have arranged to have it taught entirely by demonstration.

It has been further agreed to institute a chair in this school, from which will be taught the *elements*, or rather the *principles*, of *surgery*. It is not to be expected, nor is it intended that the lecturer will here, in the short course to which he is confined, enter at length, or in detail, into surgical science. But he will be enabled to carry out, nevertheless, a very important intention. He will lay down such instruction and practice as will give the industrious man an insight into the higher practical branches of surgical skill, and will make, as the learned Caphagus has it, "the rudimans" so clear that no difficulties shall stand in the way of him who may choose to apply—after observation and reading, to the mastery of the science in its full breadth and usefulness.

The introduction of a chair of *chemistry* into an educational establishment of this kind carries with it a practical, as well as a philosophical meaning. In the scientific family chemistry is the industrious sister, who never gets married, who has always more work of her own than she can do, and who is withal inexhaustible in the assistance she can render to the members of her circle, from brothers and sisters direct to nephews and nieces of all generations.

It matters little where, in the history of the world, we pick up this science, whether we trace it back to Tubal Cain, *malleator et faber;* to the Egyptian Hermes and his magical expositors, known equally to Moses or Democritus; to *the* chemist—*i. e.*, alchemist, and his transmutations of vile metals into gold; to Paracelsus, with his immortality-giving draught; to his rhapsodic followers, the Rosicrucians; to Agricola, seeking wisely to turn chemistry to application in things of common life; to Berner and Becher, systematising the whole crude form into philosophy; or to Mahow and Stahl, penciling out the chemistry of living things: to whichsoever origin, I say, we trace back the science, we find it exerting an influence century by century, year by year, month by month, and now week by week increasing, until at length it has infused itself into all that we know and most that we do. But of late years this pervading science has certainly taken advance in no one direction more rapidly and more advantageously than in that into which it was first turned by the far-famed Agricola—I mean its application to the daily wants and arts of life. To chemistry in this sense the eye of every man is turned. For this the northern artisan, with dye-stained hand, stipulates with his employer for one hour a day, that he may listen to his chemical professor. For this the Prince of Wales, and hope of England, leaves the realm of royalty to learn the secrets that have made his coun try so great and himself, through his country, so important in the eyes of all the world. Under various unnecessary names, such as metallurgy and technology, the science has thus been carried into new shades and grades. But, under whatever names, these, in an elementary sense, all mean, chemistry in its practical application to the wants of man.

Now, in regard to dentistry, chemistry comes in with all her force, use, and friendliness. She tells the dentist, and she will tell him much more if he will only listen to her, what metals will best mould themselves to his will; what amalgams in respect of color, durability, and harmlessness, will answer his purposes best, and what materials will supply the most convenient substitutes for natural parts which have succumbed to disease. Nor, in relation to dentistry, does she stop here. In the knowledge she supplies as to the effects of external agents on organized structures, and of animal secretions on animal textures, she suggests much as to the first causes of natural disease, and the means by which such natural disease may be either prevented or cured. Lastly, it is the office of chemistry to teach the elementary nature of things, organic as well as inorganic. To show of what parts each living structure is composed—tooth and bone, nerve and muscle; to indicate the method in which these parts range themselves into compounds and structures, and to compare arrangements in healthy and diseased states. In these respects, therefore, she assists materially the understanding in its appreciation of the laws of physiology or pathology, in their unity and in their combination.

If I have endeavored thus to render the good science-sister a good turn, with some warmth of commendation, I have but done so on the principle that "one good turn deserves another;" for to me she has rendered much, and, by that sympathy which binds men of scientific taste in one bond, I feel that in offering the privileges of a chemical education, however short to the industrious student in dentistry, there is held out to him, both in poetry and practice, a pursuit which, followed out honestly, gives a daily recompense.

In the design of the school one last, but not least, object is brought into view. We have thought it desirable to introduce comparative anatomy into the plan. We have a reason for this, and a valid one. I have already stated that it is our intention not only to supply the necessaries, but to supply such further knowledge as shall form a nucleus for pure science, to which the wearied man may turn and feel in his relievement a mental occupation, and therewith a novelty, a pleasure, and a treasure. Now, there is a curious psychological fact as regards professions; they, like men, exhibit individually their special tendencies to particular tastes and pleasures, towards which, either by habit or nature, they turn as it were irresistibly, and sometimes show a genius which would not otherwise have been brought out. With regard to dentistry and its representatives, this disposition is seen leaning to natural history. I do not mean to flatter and say that the pursuit of natural history is as yet intimately allied with dentistry, but I say that the tendency to such alliance is very clearly marked in all parts of the world where dentistry is practiced; for, not only have many dentists, such as Nasmyth, George, and Bell, shone in this department of natural science, but the greatest comparative anatomist or natural historian of his day, John Hunter, contributed largely to dentistry, and seemed, out of the range of his philosophical thoughts, to hold the study of the teeth in the first rank as a practical pursuit.

Seeing, then, this natural tendency, we have determined to encourage it in our efforts, to suggest, in an outline of this science, the taste for its better acquaintance, to supply the principles, and, in regard to the comparative anatomy of the structures, which more particularly concern the practitioners adduced, to supply the details in their richness.

Did it belong to me to hold the pen of St. Pierre, that so watching with him through his microscope the architecture of an insect city, built on a mere strawberry leaf, I could, from the height of the greatness of man above insect, depict what I can only borrow, the outline of that microcosm; the city with its naturally constituted rafters of gold, pillars of ivory, and arches set with ruby and topaz; its cups, urns, pavilions, and domes, which human architect and goldsmith have not rivalled. Could I depict, in continuance of the brilliant history, the beings that live under a reflex thus enriched to whose perception through their compound eyes a drop of dew filtering in a capillary transparent tube is as a thousand cascades; or could I, leaving this tiny natural world, carry you with the force of Cuvier or Humboldt to the description of that leviathan which those who go down to the sea in ships see and wonder at; could I describe his habits and home, I might be expected, nay I might, from the two extremes of great and little, and their connecting links, sketch out in graphic force a picture at which the imagination should faint, first to behold and then to comprehend. To me this knowledge, this power belongs not. I can but offer to the student such childish thought for his ambition as invests a midsummer dream when something is said or suggested, for which more is waited, and which afterwards, undefined and undefinable, excites yet to desire and hope—the desire, the hope of some realization as substantial as it is unknown, and which, having neither shape, nor color, nor form, is nevertheless felt to be radiant with beauty.

It will be the congenial, the delightful task of one of my colleagues to lead the student through the range of truth above shadowed forth, nor, despite the tenth great precept, can I envy him less in his labors than admire him for the ability and industry by which those labors are sure to be rewarded.

But when I have pointed out the indirect value of the natural history pursuits, I have scarcely given to them even fair commendation, or their just weight. In them is embodied something also of the direct and the practical. By them comparative pathology is introduced. By them the structure of animal tissues and their application to the uses of man are made apparent. By them the analogies between man and his lower earthmates are established.

I have thus placed forward the intentions, in so far as I can represent them, of the founders of this school. It has been so long the custom, in considering the question of professional schools, to connect with them the idea of an infinitude of departments, that the staff of teachers may at first sight seem capable of increase. We hope, however, to make it obvious in practice, that limitation of subjects instead of proving an inconvenience, by obliging us to group analogous studies round a common centre, and to reduce the actual amount of personal attendance on the part of the student will cause the unities of natural science to be more clearly depicted, and labor to be conserved. If there has arisen one doubt on these points, it refers to the subject of *materia medica*, as a department in itself distinct. It has been intimated to the faculty of lecturers, that as there are certain remedial agents used in dentistry —in the way of lotions, balsams, stoppings, and the like— it had been well to make this a particular study. The suggestion, after having undergone careful consideration, has not been adopted. The faculty has concluded, that all reference to such remedial measures belongs naturally to the lecturer on dental surgery, who will therefore make it a special part of his course.

I have spoken in so far on matters pertaining to the Metropolitan School of Dental Science, as a separate institution it is incumbent on me, however, to allude to two other facts, which extraneous in themselves, have nevertheless a bearing on the success of the school as an educational enterprise.

Firstly, then, in order that the students here educated may have the advantage of clinical instruction under teachers, some of whom are also teachers in the school, the dental practice of the Westminster Dispensary has been rendered, by new and excellent arrangements, subservient to practical education. Here the student for a small fee may enter to a special course of learning, and may supplement the school studies by daily observation and personal experience.

In the second place, the College of Dentists, by a recent change in its laws, has established an examination for membership. With sound discretion, the college authorities have enforced no curriculum of study. But as their examination requires knowledge, and knowledge which must be obtained, the opening of a school at the present moment stands in the free-trade relation of supply to demand.

And here the opportunity is most fitting for an acknowledgment, on the part of the faculty of lecturers, of the kind interest and valuable assistance rendered to the faculty by the council of the college. Not only have the members of the council exerted themselves individually for the success of the school, but in their united and official capacities they have granted an efficient aid, without which the school itself had scarcely been established—or at least not without an excessive outlay of labor and of pecuniary means.

When I state that excellent accommodation for lecture purposes has been allowed to the faculty at a mere nominal rent ; and that three valuable prizes (one of them named in honor of the founder of the college the Rymer prize) have been offered as rewards to those students who shall most distinguish themselves in industry and proficiency, I have said sufficient to indicate the liberality of the council of the college, and its earnestness in the cause of professional education.

In return it has been a source of equal pleasure to the faculty to offer to the college members such advantages for attendance on the lectures as shall afford a partial but grateful compensation to so much considerate care and friendly assistance; both the more acceptable because devoid of pretence and strictly in season.

From the history of the school and its intention, I pass now from a general to a particular topic—to the selection of a few phrases addressed purely to the student, his labors and his prospects.

I need not repeat what has been said as to the conditions under which the student life may be commenced in this place, nor as to the class of students for whom the instruction is intended. I shall assume at once that my words reach only the ears of the industrious and well-meaning, so that in the way of direction and encouragement there will be required no line from me as to moral conduct or example in life. I want, indeed, only to speak of the way to work, and of the rewards which are offered for work well done.

The primary principle, then, I would lay down as necessary to success is *reliance.* Reliance not so much in teachers and books as in the inner self, as in the power of the individual mind to doing and conquering. The student resting on his own strength for action, and on the strength of others more advanced for the supply of knowledge, is already more than half-way on his road to position and honor. In suggesting this reliance, understand that I do not mean by it conceit. Conceit is not reliance on self, but want of reliance in every thing that is not self. Conceit is therefore, in truth, a real want of self-reliance; since man is so constituted that his own faith, however great, must, to be applied, be linked to a certain degree, with the faith that is in others.

No! I mean by self-reliance such simple trust in industry, in thought, in natural capacity, as shall enable the student to feel that he can work as others work, learn as others learn, and succeed as the best. Or, to put the principle in another light, I mean a negation of doubt, the parent at once of idleness and of failure. The poet has indeed well instructed us when he says—

"Our doubts are traitors,
And make us lose the good we oft might win,
By fearing to attempt."

And the prose poet Bunyan, when he puts his hero into Doubting Castle, expresses with another intention the same truth. Therefore I commend you to steer clear of doubt in the acquirement of knowledge, for by this you will obtain the first great secret of learning—simple trust in self and in learning itself.

But the negation of doubt, or in other words, the appreciation of faith as to your own powers, must, to be kept in order, be kept in work, and must be based on the constant and repeated observation of the natural phenomena which it may be your duty to investigate. This thought leads me to the naming of another faculty of mind as an essential one to be cultivated. I will call it *solidity*, as the best synonym for a long definition.

Now solidity is obtainable in study by this preliminary process, a determination at first to learn all facts and no opinions. In natural studies the possibility of this resolve is easy, because the natural laws and ordinances, in so far at all events as we can comprehend them, are uniform and stable; or so nearly so that trifling aberrations in details serve only to imprint more firmly on the mind the elementary truth. You, therefore, setting forth in your pursuits with a certainty of precise knowledge before you, may, without the least fear, throw your whole energies into your labor, with the conviction pre-eminent that by constant and close attention to each fact, and by repetition and repetition, you must learn and learn well. Leave you this system in your early days, and the smoothness of your course is lost. The apparent true will melt into the absolute false, and the laughing world will drive even hope out of your hearts.

When the great Columbus set out from Palos to sail into an unknown sea, his companions not appreciating the primary facts on which the faith of their commander was founded, saw ever and anon in the horizon island upon island; they defined the shores, counted the mountains, shaped the bays, and even traced the rivers. These islands their master could never see; and as the little caravels steered on their way the islands melted (for they were cloud islands,) melted into the thin air. The sailors of Columbus are but the types of those students entering into the domain of natural science, who, fixing their vision on cloud islands and suffering grievous disappointment as island after island dissolves, lose their self-reliance, lament the day on which they set forth on their wanderings, and offer open mutiny to those who would guide them safely to the goal. I beg you, then, as I wish for your prosperity, to remember this simile, and to be mindful so to adhere in the early part of your careers to the cultivation of positive knowledge, that if it ever be your fate to venture into the open sea, you, like Columbus, may have such a foundation of abstract truth to rest upon as shall carry you stout of heart and reliant until the land of your discovery is in view.

But you will ask me how you are to know the real from its similitude. The secret is simple. Learn as fact all that is demonstrable to your senses. You may have heard that in studies such as yours, there is little pretence to demonstration. The insinuation is untrue. You will have to learn anatomy—human and comparative. That carries with it direct demonstration. You will learn physiology; the best of that admits of direct demonstration. You will learn operative science; that may be exact in its teachings. You will learn the physical properties of elements and compounds; and here too the demonstrable is the possible. There is in short, in all these sciences, enough to be acquired in positive knowledge to occupy the first five years in the life of any student. If I were asked, indeed, to write a library of science for educational purposes, I would throw all present systems aside, and begin each series with a lesson-book, showing what in that department has been actually proved. As there are no such ready method at hand, you must grub out the system for yourselves—a task you will readily perform if you remember the simple rule, that a natural fact is always a demonstration.

At the same time, bear strictly in mind that it is by no means necessary to place a low estimate on the advancement of knowledge by logical theory ; nor am I desirous to prevent you from the pursuit of first-class theoretical learning. The highest genius of men of all sciences has exhibited itself in the construction of theory, and much interesting knowledge is based on nothing else. I wish you simply to let alone the theoretical until you have grasped the actual. This achieved, the further wish is mine, that the genius of Newton for theoretical contemplation may be yours.

Another mental faculty, which I would have you cultivate, is that of *learning by gradation*, step by step from fact to fact, from mastery of facts to their combinations, and from thence to the study of theory, as placed on these facts, single or combined. On this point, the admirable Locke, whose essay "*on the conduct of the understanding*" should be bought by you all as the first of class-books, has an observation which in its depth and simplicity has never been surpassed. Thus it runs :—

"The surest way for a learner in this, as in all other cases, is not to advance by jumps and large strides ; let that which he sets himself to learn next, be indeed, the next, *i.e.*, as nearly conjoined with what he knows already as is possible : let it be distinct but not remote from it, let it be new, and what he did not know before, that the understanding may advance ; but let it be as little at once as may be, that its advances may be clear and sure. This distinct gradual growth in knowledge carries its own light with it in every step of its progression in an easy and orderly train, than which there is nothing of more use to the understanding. And though perhaps this may seem a very slow and lingering way to knowledge, yet I dare con fidently affirm whoever will try it in himself, or any one he will teach, he shall find the advances greater in this method, than they would, in the same space of time, have been in any other he could have taken. For the greatest part of true knowledge lies in a distinct perception of things in themselves distinct."

I should spoil the whole effect of my proposition, were I to attempt to qualify or to add to so masterly and clear an exposure of its truth and its promises.

A last principle which I would suggest to you as to be remembered in your philosophical pursuits, I shall designate *concord*. We may consider this principle as bearing with it two motives, as applying, that is to say, to one's self and one's own studies, and to others and to their proceedings. As applying to your own pursuits, it simplifies them, and strengthens their realities. As in music the word concord suggests two distinct notes, which struck together make a new and perfect sound, as well as an infinitude of such combinations, all in harmony; so in the other sciences the word conveys to the educated mind the unities of science, and the modifications, however endless, which are built of the unities. It is indeed by knowledge of this concord, that the man of true genius is separated from the man of ordinary acquirements.

The latter is always doing the drudgery of detail, learning individual intervals and discovering more. The former puts these together and finds the combinations. The one makes the instrument, the second writes the inspiration. They may both be useful in their way, but I would have you of the first.

But the word concord expresses an idea of more common-place character, but not less useful to us as men. It recalls to the classical mind "Concordea," and her temple by Camilius. It conveys in this sense the lesson that, for the proper pursuit of science, concord with other minds is necessary. A difficult suggestion this, I know, one hardest of all to carry out, for mind is the most unstable attribute in nature. But if there is one means more than other by which, in your relations with your fellows, moral concord is to be sustained, it is this: That you mark out your own business and mind that; and that, if you have anything to say original on your part, you say it with care and modesty, and leave it there as it has been spoken, content to hold to it, to modify it, or to withdraw it, as time and truth shall show its worth, but entering into no petty war as to its merits, and wearying no one by protestations as to its importance.

I press these commendations earnestly on your minds. The time, not less than the opportunity, bids me to the act. There is now extant in science a class of men by whom science herself is stripped, and by whose clamor she is shaken to her centre. This class of men, having in themselves no ardent love for nature, having no true ambition, having neither courage nor industry for personal work, and having no method in their lives, seek notoriety, seek position, by systematically lying in wait to entrap and crush independent labor and individual exertion. With the least pretension to be the critics' work these become the critics of the day, and as they learn the arts of supplanting logic by plausibilty, and fact by perversion of it, they gain unquestionably a temporary influence, which brings to the true and honest heart the most embarrassing of all difficulties. You will meet these difficulties best by avoiding all entanglement with them. By yourselves never assuming the position of arbitrator; and by treating every critical word against your own well-intentioned and, to your best ability, truthful work, in one unanswerable argument—*absolute silence.*

I might, perchance, extend these observations as to the mode and advancement of learning; but if in that which has preceded the four cardinal principles—*self-reliance, solidity, gradation,* and *concord*—have been enforced with any success, the rest or the sequential may well wait.

It remains now for me but to point out the rewards which follow the pursuits of natural and professional knowledge.

It were unnecessary for me to denote the value of knowledge in the abstract, or to labor at elaborating a sentiment long since expressed in three words, "Knowledge is power." Yet I must for a moment rest to observe that, to the younger members of the profession I address, there are certain particular and substantial advantages to be derived from it worthy of their most sanguine and purest hopes. There are before them for competition immediate honors. There are the ultimate rewards—an advancing social status, a certainty and precision in practice—a superiority in thought, manner and act,—the appreciation of the public and the road to scientific fame.

Speaking as a scientific man, I know of no class to whom the honors of science are so freely offered as to the dentist. His occupation, at once professional and remunerative, permits of quiet for the most contemplative studies. The medical man, with a load of responsibilities ever on his shoulders, with human life resting on his skill, and anxious friends hanging on his lips for hope, has some excuse if, in the active practice of his special science, he leaves the general. The dentist, having no such responsibilities, has no such excuse ; and if, with present advantages before him, he allows medicine still to take the lead in general learning, his supineness is unpardonable.

But, gentlemen, I should fail sadly in completing this day's most refreshing task did I not point out to the student, now for the first time entering his new life, that above and beyond all I have promised him as the reward of his exertions there is a result derivable from them which neither fortune can modify, nor time remove, nor death destroy. I mean that in the studies with which he will grow conversant there is implied a communion with the good and the beautiful, which calms passion, subdues pride, and fills the mind with the ever-constant impression that what is must remain, and that an universal essence from which we are none of us removed, holds all creation in one common bond, and by an universal sympathy.

There is an ancient fable that Orphæus, or Philosophy, going into solemn solitude, by one magic touch of his lyre drew to him natures of all kinds, and by the sweetness of his melody made them as one in general harmony of feeling, and in the appreciation of the influence which so charmed their lives. There is hidden in this exquisite fable more than at first appears. It represents in figurative terms the harmony of nature and the all-pervading influence exerted on intelligent beings by that harmony in its fulness.

Beautifully, forcibly, is the lesson here taught, but weak when compared with that modern and less obscure expression, which explains in direct terms that the elevating and combining influences of natural pursuits rest on the fact that they by necessity, in proportion as we cultivate them, lead us nearer and nearer to the beginning and end of all.

Shall I hesitate at a sentence? or tremble over a thought that may surpass the Grecian fable? I need not. The poet physician Akenside, in one paragraph, redundant of all I could say, lends me this last impression to leave with you:

"The men whom nature's works can please
With God himself hold converse. Grow familiar
Day by day with his conceptions, act upon his plans,
And form to His the relish of their souls."

[*London Dent. Rev.*

